# Spontaneous Regression of a Middle Ear Melanoma

**DOI:** 10.1097/MAO.0000000000003371

**Published:** 2021-09-30

**Authors:** Iris Krebbers, Henricus P. M. Kunst, Laura W. J. Baijens, Mari F. C. M. van den Hout, Jerome J. Waterval

**Affiliations:** ∗ Department of Otorhinolaryngology and Head & Neck Surgery, Academic Alliance Skullbase Pathology MUMC+ - Radboudumc Maastricht University Medical Center, Maastricht, The Netherlands; † School for Oncology and Developmental Biology – GROW, Maastricht University Medical Center, Maastricht, The Netherlands; ‡ Department of Otorhinolaryngology and Head & Neck Surgery, Academic Alliance Skullbase Pathology MUMC+ - Radboudumc, Radboud University Medical Center, Nijmegen, The Netherlands; § School for Mental Health and Neuroscience, Maastricht University Medical Center, Maastricht, The Netherlands; || Department of Pathology, Maastricht University Medical Center, Maastricht, The Netherlands

**Keywords:** Melanoma, Middle ear, Neoplasm regression, Spontaneous

## Abstract

To describe a case of complete spontaneous regression of a middle ear melanoma.

**Patient:**

We present a case of a 68-year-old man with complaints of unilateral hearing loss and an ipsilateral facial nerve paresis. Radiological and histopathological examination revealed a cT4bN0M0 mucosal melanoma of the middle ear.

**Interventions:**

The patient underwent a subtotal petrosectomy and postoperative radiotherapy.

**Main Outcome Measure:**

Computed tomography (CT), magnetic resonance imaging (MRI), positron emission tomography/computed tomography with 2-[fluorine-18]-fluoro-2-deoxy-D-glucose (FDG-PET-CT), and histopathological examination.

**Results:**

After subtotal petrosectomy, histopathological examination of the resection specimen showed only fibrosis and a histiocytic and clonal T-cell infiltration, but no residual melanoma at the primary tumor site, consistent with spontaneous tumor regression. Follow-up MRI scanning 6 and 12 months after radiotherapy showed no signs of tumor recurrence.

**Conclusions:**

This case describes the concept of spontaneous regression of a mucosal melanoma of the middle ear. Spontaneous tumor regression at this location has not been described before.

## CLINICAL CASE

**FIG. 1:**
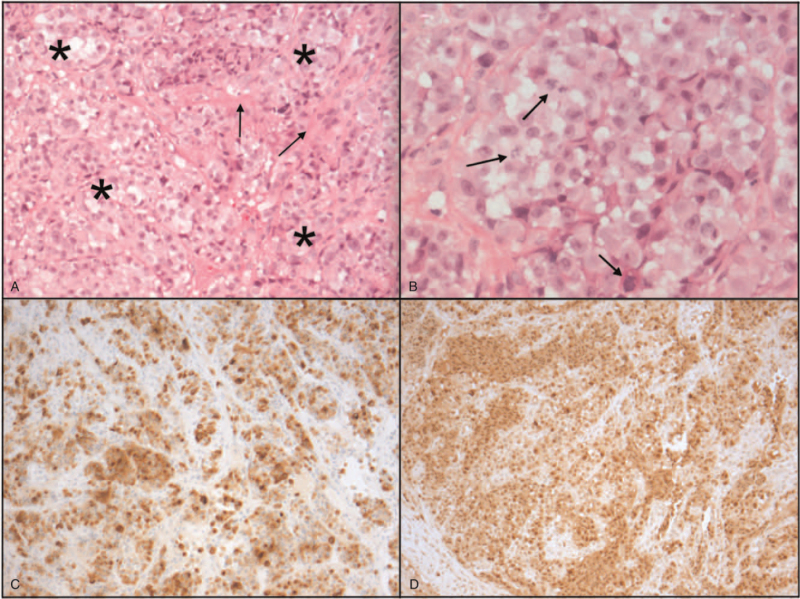
*A*, Representative images of the biopsy specimen of the middle ear melanoma: hematoxylin-eosin staining (200×) shows nests and sheets of a highly atypical melanocytic proliferation (^*∗*^) within a fibrous background (*arrows*). *B*, Detail (hematoxylin-eosin, 400×) of the proliferation shows that tumor cells have an epithelioid, eosinophilic, partly vacuolated cytoplasm with anisomorphic nuclei with macronucleoli and prominent mitotic activity (*arrows*). *C*–*D*, The cell population is positive for Melan A and S100 stains (in brown).

A 68-year-old male patient was referred to the department of otorhinolaryngology because of left-sided, progressive hearing loss. He was suspected of having an otitis media with effusion. However, routine clinical examination after 3 months showed an atypical granulomatous presentation of the tympanic membrane and therefore a biopsy was taken. Histopathological examination showed a highly atypical and mitotically active stromal melanocytic proliferation, positive for S100, Melan-A, and MITF (Fig. 1A–D), consistent with the diagnosis of a mucosal melanoma. Subsequently the patient was referred to our institute.

One week after biopsy, the patient visited our outpatient clinic. At that moment the unilateral hearing loss had increased and an ipsilateral facial nerve paresis (House–Brackmann [HB] V/VI) was almost in remission. The patient had no complaints of otalgia or otorrhea and there was no history of an ear trauma. He had a history of hypertension, atrial fibrillation, coronary artery disease, depression, and obstructive sleep apnea syndrome. There was no family history of melanoma. At physical examination, inspection of the facial musculature showed a mild degree of asymmetry of lip closure (HB II/VI). Otomicroscopy showed a mass at the level of a seemingly thickened (or absent) tympanic membrane with blue discoloration at the posterior upper and lower quadrant post-biopsy. This mass was non-pulsatile and the usual anatomical landmarks such as the annulus fibrosus tympanicus and the tympanic membrane could not be identified. The Rinne test using a 512-Hz tuning fork was negative on the left side and the Weber test was lateralized to the left ear. Further physical examination including nasal endoscopy and palpation of the neck showed no abnormalities. The audiometry examination showed a left-sided mixed hearing loss of 92 dB HL with maximum speech recognition of 88% at 110 dB SPL.

**FIG. 2:**
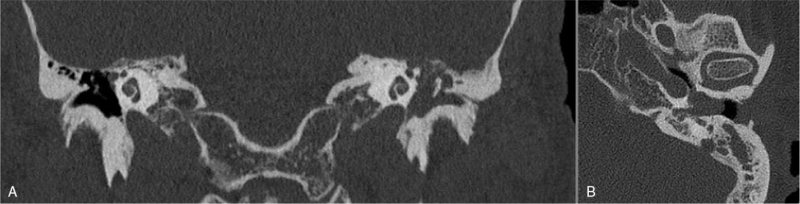
Computed tomography (coronal [*A*] and axial planes [*B*]) revealed soft tissue invasion of the left mastoid bone and the middle ear cavity, completely embedding the ossicular chain.

Computed tomography (CT) and magnetic resonance imaging (MRI) revealed soft tissue invasion of the left mastoid bone and the middle ear cavity, completely embedding the ossicular chain (Fig. 2). Dehiscence of the bony facial nerve canal, tegmen tympani, and middle cranial fossa plate with contrast enhancement of soft tissue protruding through these defects, was observed. An ultrasound of the neck was performed and revealed an enlarged left-sided neck node. Fine needle aspiration cytology showed reactive lymphoid cells without melanoma.

**FIG. 3:**
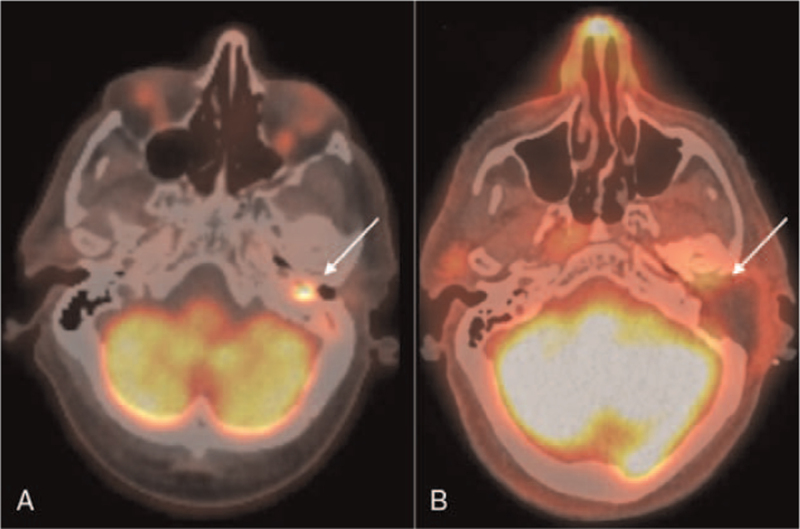
*A*, Positron emission tomography/computed tomography with 2-[fluorine-18]-fluoro-2-deoxy-D-glucose (FDG-PET-CT) demonstrated intense FDG-uptake at the left middle ear cavity indicative for a malignant tumor. Only moderate FDG uptake was seen at the level of the external auditory canal and the mastoid bone. *B*, One month after surgery a FDG-PET-CT follow-up scan revealed minimal soft tissue induration at the petrosectomy site and mild FDG uptake with no evidence of residual tumor.

Positron emission tomography/computed tomography with 2-[fluorine-18]-fluoro-2-deoxy-D-glucose (FDG-PET-CT) demonstrated intense FDG-uptake at the left middle ear cavity indicative for a malignant tumor based on the histopathological diagnosis (Fig. 3A). Only moderate FDG uptake was seen at the level of the external auditory canal and the mastoid bone. There was no evidence of regional or distant disease.

The tumor was staged according to the 8th TNM Classification for Mucosal Melanoma of the Head and Neck as a cT4bN0M0 malignant mucosal melanoma originating from the left middle ear cavity (1).

One month after presentation at our outpatient clinic, a subtotal petrosectomy was performed. During surgery, suspicious brown-colored granulation tissue was obtained from the middle ear cavity and mastoid bone. Furthermore, several defects of the tegmen tympani were present at the level of the middle and posterior cranial fossa. The granulation tissue was removed from the mesotympanum, hypotympanum, sinus tympani, oval window, antrum, mastoid cavity, and perilabyrinthine cell tracts. Microscopically radical resection of the aberrant tissue was not possible due to the extensive spread. The middle ear cleft was obliterated with abdominal fat.

**FIG. 4:**
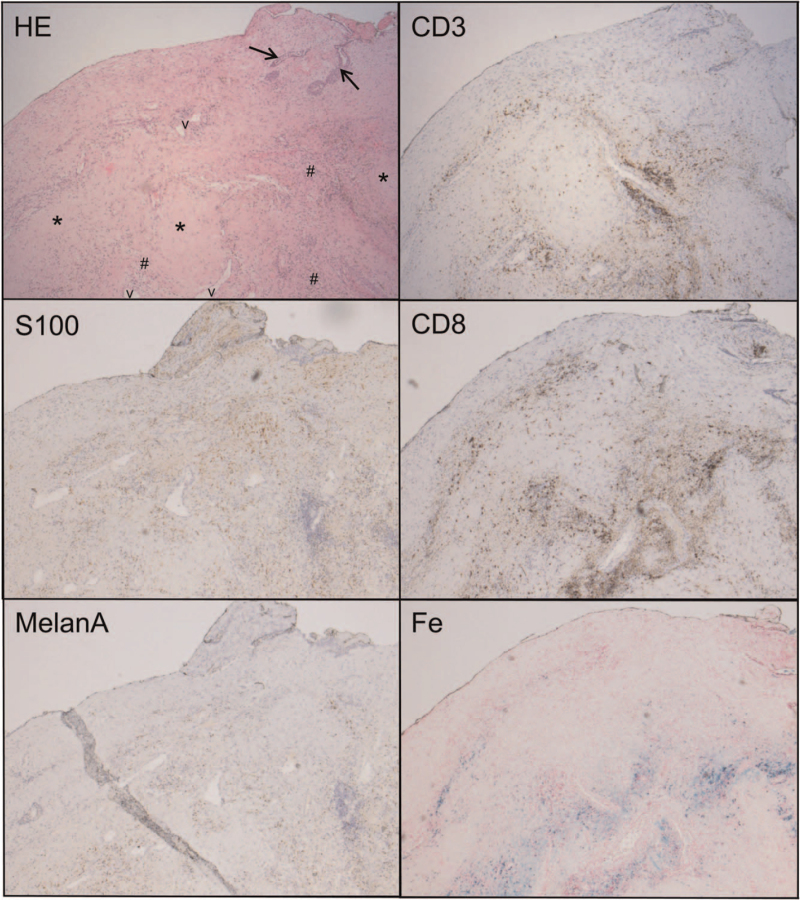
Representative images from the petrosectomy specimen showing scar-like tissue with fibrosis (*∗*), dilated blood vessels (*v*) and a lymphohistiocytic cell infiltration (*#*). In the right upper quadrant (hematoxylin-eosin [HE] staining) residual middle ear epithelium is seen (*arrows*). S100 stains histiocytic and dendritic-type cells in brown, no residual melanoma cells. Melan A is negative (brown background pigments are iron deposits). CD3 shows the T-cell infiltration, which is partly CD8-positive (*brown dots*). Perls (Fe) staining shows the iron deposits in blue corresponding to the intraoperatively described brown-colored tissue.

Histopathologically, the resection specimen was completely blocked and numerous additional levels were made, but no residual melanoma cells could be found. However, a moderately dense CD3^+^, partly CD4^+^, partly CD8^+^ T-cell infiltrate, and CD68^+^ histiocytic infiltrate was observed within a large area of fibrosis and dilated blood vessels. Additional melanocytic stainings (Melan-A, S100, and SOX10) were all negative (Fig. 4). The area of fibrosis was consistent with the previous biopsy site. These histopathological findings are in accordance with spontaneous regression of the mucosal melanoma (2). Furthermore, additional T-cell antigen receptor (TCRγ and TCRβ) clonality assays (PCR-based according to Euroclonality, BIOMED) showed a highly clonal T-cell response. To rule out accidental mix-up of samples, microsatellite examination was performed on the biopsy and the resection specimen, confirming that both specimens were from the same patient.

One month after surgery, the facial nerve paresis was in complete remission (HB I/VI) and a FDG-PET-CT follow-up scan revealed minimal soft tissue induration at the petrosectomy site and mild FDG uptake with no evidence of residual tumor (Fig. 3B). Since microscopically radical resection was not possible, postoperative radiation therapy was discussed with the patient. The patient received postoperative local radiation therapy, 66 Gray in 33 fractions. Follow-up MRI scanning 6 and 12 months after radiotherapy showed no signs of tumor recurrence, regional or distant disease. Unfortunately, the patient died 13 months after treatment from an acute myocardial infarction.

## DISCUSSION

Malignant melanoma develops through the neoplastic transformation of pigment-producing cells, usually located in the epidermis and dermis (2). Accumulation of mutations occurs in genes responsible for cell proliferation and apoptosis. Melanocytes are derived from neural crest cells and therefore, melanomas can develop in various places in the body (3,4). Although melanomas are usually cutaneous in nature, they can also occur in various extracutaneous sites including ocular, mucosal, and leptomeningeal locations (4–6). Mucosal melanomas are rare and represent 1.4% of all melanomas (7,8). Mucosal melanomas arise from mucosal epithelium anywhere in the respiratory, gastrointestinal and genitourinary tracts (7). Head and neck mucosal melanomas are predominantly found in the sinonasal region and oral cavity (6,7). This is usually an aggressive form of cancer without symptoms in the early stages because of these “hidden” sites, resulting in late diagnosis and poor prognosis (6). The main function of melanocytes is pigmentation and UV protection of the skin and the eyes. However, melanocytes are also present at sun-protected mucosal areas of the body where they are also believed to play a role in the immune system because of their phagocytic, antigen-presenting, and cytokine-producing properties (6). Primary mucosal melanomas of the head and neck are uncommon and because of their rareness, knowledge on their pathogenesis and prognosis is scarce (7).

In the present case, no residual melanoma cells were found in the petrosectomy specimen. Regression of malignant melanoma is histologically characterized by the disappearance of neoplastic melanocytes which can occur spontaneously or in response to treatment (2). Histological regression of a primary melanoma can be partial, segmental, or complete and is more common in melanomas compared with other types of tumors (4,5). It is estimated that about 50% of non-metastatic melanomas (mucosal melanoma included) partly regress spontaneously (2). Spontaneous regression in metastatic melanomas is rare and the pathophysiology of regression of mucosal melanoma is not completely understood. The majority of spontaneous regression studies in mucosal melanoma refer to the cutaneous type and suggest that it may be caused by an interaction between melanoma cells and the host immune system, resulting in killing of melanoma cells, proliferating lymphocytes, telangiectasia, and replacement of tumor tissue by fibrosis (2,5,9,10). Various clinical signs of spontaneous regression have been described such as hypopigmentation, telangiectasia, size reduction, and scarring (2,5). The intraoperatively described suspicious “brown-colored granulation tissue” was histopathologically based on fibrotic scar tissue with inflammation, a macrophage clean-up response and iron deposition explaining the discoloration. Besides immunological and inflammatory factors, blood transfusion, pregnancy, and endocrinological conditions such as diabetes mellitus have been associated with spontaneous regression in the literature (2,5,11). Our patient was not known with any of these conditions. It has also been suggested that infection and surgery elicit an increased host immune response directed against specific melanocyte-associated antigens such as Melan-A (5,11,12). Furthermore, surgical intervention may lead to an interruption of blood supply resulting in disintegration of the tumor (11). In this context, one could hypothesize that the previous biopsy of our patient elicited an immune reaction. Spontaneous regression is thought to result from an effective immune response with CD4^+^ and CD8^+^ T-cells directed against melanoma cells (2). These infiltrating T-cells have a clonal T-cell receptor profile. In our case, a CD4^+^, CD8^+^ T-cell, and CD68^+^ histiocytic infiltration was present within a large area of fibrosis in the petrosectomy specimen. These histopathological findings were present in an area consistent with the previous biopsy site, supporting spontaneous regression of mucosal melanoma. Additional T-cell antigen receptor clonality assays (EuroClonality/BIOMED-2 Ig/TCR) performed on tissue from this area showed a highly clonal T-cell response, further supporting the phenomenon of regression (13).

Few cases of primary middle ear mucosal melanomas (n = 12) have been reported in the literature (9,10,14). Oliveira et al. (9) describes a case of spontaneous regression of an oral mucosal melanoma in a patient who refused treatment with no signs of tumor recurrence 6 years after treatment. Patients from the other reports underwent multimodality treatment such as surgery followed by radiotherapy and/or systemic therapy. Middle ear melanoma was accompanied by poor outcomes showing 20% local recurrence, 50% distant disease, and a 70% mortality rate (10,14). Spontaneous tumor regression at this location has not been described before.

## References

[1] Huang SH O'Sullivan B. Overview of the 8th Edition TNM classification for head and neck cancer. *Curr Treat Options Oncol* 2017; 18:40. 28555375 10.1007/s11864-017-0484-y

[2] Cervinkova M Kucerova P Cizkova J. Spontaneous regression of malignant melanoma - is it based on the interplay between host immune system and melanoma antigens? *Anticancer Drugs* 2017; 28:819–830. 28609309 10.1097/CAD.0000000000000526

[3] Zito PM, Scharf R. Cancer, Melanoma of the Head and Neck. StatPearls, 2019. Available at: https://www.ncbi.nlm.nih.gov/books/NBK513248/. Accessed August 1, 2020.

[4] Mehta BS Maniyar H. Primary mucosal malignant melanoma of middle ear - a case report. *Indian J Otolaryngol Head Neck Surg* 2007; 59:71–72. 23120394 10.1007/s12070-007-0022-5 PMC3451738

[5] Bramhall RJ Mahady K Peach AHS. Spontaneous regression of metastatic melanoma. Clinical evidence of the abscopal effect. *Eur J Surg Oncol* 2014; 40:34–41. 24139999 10.1016/j.ejso.2013.09.026

[6] Mihajlovic M Vlajkovic S Jovanovic P. Primary mucosal melanomas: a comprehensive review. *Int J Clin Exp Pathol* 2012; 5:739–753. 23071856 PMC3466987

[7] Tyrrell H Payne M. Combatting mucosal melanoma: recent advances and future perspectives. *Melanoma Manag* 2018; 8:MMT11. 10.2217/mmt-2018-0003 PMC6240847 30459941

[8] McLaughlin CC Wu XC Jemal A. Incidence of noncutaneous melanomas in the U.S. *Cancer* 2005; 1:1000–1007. 10.1002/cncr.20866 15651058

[9] Oliveira AA Ching L Teixeira GY. Spontaneous regression of oral mucosal malignant melanoma. *Arch Head Neck Surg* 2020; 49:e00162020.

[10] Rajan R Samant S. Spontaneous regression of mucosal melanoma. *Otol Head Neck Surg* 2008; 139:128.

[11] Kalialis LV Drzewiecki KT Klyver H. Spontaneous regression of metastases from melanoma: review of the literature. *Melanoma Res* 2009; 19:275–282. 19633580 10.1097/CMR.0b013e32832eabd5

[12] Aung PP Nagarajan P Prieto VG. Regression in primary cutaneous melanoma: etiopathogenesis and clinical significance. *Lab Invest* 2017; 97:657–668. 10.1038/labinvest.2017.8 28240749

[13] Langerak AW Groenen PJTA Brüggemann M. EuroClonality/BIOMED-2 guidelines for interpretation and reporting of Ig/TCR clonality testing in suspected lymphoproliferations. *Leukemia* 2012; 26:2159–2171. 22918122 10.1038/leu.2012.246 PMC3469789

[14] Maxwell AK Takeda H Gubbels SP. Primary middle ear mucosal melanoma: case report and comprehensive literature review of 21 cases of primary middle ear and eustachian tube melanoma. *Ann Otol Rhinol Laryngol* 2018; 127:856–863. 30103615 10.1177/0003489418793154

